# The Effect of Collaborative Reviews of Electronic Patient-Reported Outcomes on the Congruence of Patient- and Clinician-Reported Toxicity in Cancer Patients Receiving Systemic Therapy: Prospective, Multicenter, Observational Clinical Trial

**DOI:** 10.2196/29271

**Published:** 2021-08-05

**Authors:** Andreas Trojan, Nicolas Leuthold, Christoph Thomssen, Achim Rody, Thomas Winder, Andreas Jakob, Claudine Egger, Ulrike Held, Christian Jackisch

**Affiliations:** 1 OnkoZentrum Zürich Zurich Switzerland; 2 Clinic for Clinical Pharmacology and Toxicology University Hospital Zurich Zurich Switzerland; 3 Universitätsklinikum Halle (Saale) Halle Germany; 4 Klinik für Frauenheilkunde und Geburtshilfe Universitätsklinikum Schleswig-Holstein Lübeck Germany; 5 Department of Internal Medicine II Academic Teaching Hospital Feldkirch Feldkirch Austria; 6 University of Zurich Zurich Switzerland; 7 Tumor Zentrum Aarau Hirslanden Medical Center Aarau Switzerland; 8 Spital Limmattal Schlieren Switzerland; 9 Epidemiology, Biostatistics and Prevention Institute University of Zurich Zurich Switzerland; 10 Sana Klinikum Offenbach Offenbach Germany

**Keywords:** cancer, consilium, app, eHealth, ePRO, CTCAE, congruency, patient-reported, symptoms

## Abstract

**Background:**

Electronic patient-reported outcomes (ePRO) are a relatively novel form of data and have the potential to improve clinical practice for cancer patients. In this prospective, multicenter, observational clinical trial, efforts were made to demonstrate the reliability of patient-reported symptoms.

**Objective:**

The primary objective of this study was to assess the level of agreement κ between symptom ratings by physicians and patients via a shared review process in order to determine the future reliability and utility of self-reported electronic symptom monitoring.

**Methods:**

Patients receiving systemic therapy in a (neo-)adjuvant or noncurative intention setting captured ePRO for 52 symptoms over an observational period of 90 days. At 3-week intervals, randomly selected symptoms were reviewed between the patient and physician for congruency on severity of the grading of adverse events according to the Common Terminology Criteria of Adverse Events (CTCAE). The patient-physician agreement for the symptom review was assessed via Cohen kappa (κ), through which the interrater reliability was calculated. Chi-square tests were used to determine whether the patient-reported outcome was different among symptoms, types of cancer, demographics, and physicians’ experience.

**Results:**

Among the 181 patients (158 women and 23 men; median age 54.4 years), there was a fair scoring agreement (κ=0.24; 95% CI 0.16-0.33) for symptoms that were entered 2 to 4 weeks before the intended review (first rating) and a moderate agreement (κ=0.41; 95% CI 0.34-0.48) for symptoms that were entered within 1 week of the intended review (second rating). However, the level of agreement increased from moderate (first rating, κ=0.43) to substantial (second rating, κ=0.68) for common symptoms of pain, fever, diarrhea, obstipation, nausea, vomiting, and stomatitis. Similar congruency levels of ratings were found for the most frequently entered symptoms (first rating: κ=0.42; second rating: κ=0.65). The symptom with the lowest agreement was hair loss (κ=–0.05). With regard to the latency of symptom entry into the review, hardly any difference was demonstrated between symptoms that were entered from days 1 to 3 and from days 4 to 7 before the intended review (κ=0.40 vs κ=0.39, respectively). In contrast, for symptoms that were entered 15 to 21 days before the intended review, no congruency was demonstrated (κ=–0.15). Congruency levels seemed to be unrelated to the type of cancer, demographics, and physicians’ review experience.

**Conclusions:**

The shared monitoring and review of symptoms between patients and clinicians has the potential to improve the understanding of patient self-reporting. Our data indicate that the integration of ePRO into oncological clinical research and continuous clinical practice provides reliable information for self-empowerment and the timely intervention of symptoms.

**Trial Registration:**

ClinicalTrials.gov NCT03578731; https://clinicaltrials.gov/ct2/show/NCT03578731

## Introduction

Patient-reported outcomes (PRO), such as symptoms and functional status, are commonly measured in clinical trials. There is growing interest in integrating electronic PRO (ePRO) into routine clinical practice during chemotherapeutic and immunotherapeutic interventions. Most cancer patients are motivated to spend time and effort documenting symptoms during their consultation for shared reporting with physicians. Patients’ self-empowerment and self-reporting should also improve patient-clinician communication, symptom detection, and symptom control [1]. As patient experience has gained importance in regulatory decision-making, patient-reported data are increasingly being used for quality assessment and comparative effectiveness research. Mobile health solutions have the potential to improve electronic symptom documentation, and when the collection of such PRO is widely used, it facilitates communication among stakeholders [1,2]. Several apps have been designed and tested with input from patients, nurses, and physicians. These apps have gained attention and quality with respect to improving the efficacy and safety data in oncology trials and drug discovery [3-5]. Their benefits in real-world digital patient monitoring during cancer immunotherapy have been demonstrated in terms of more accurate symptom assessment, better patient-physician communication, and reduced need for telephone consultations [6-8]. As oncologists intend to share information on symptom grading with their patients, as defined by the Common Terminology Criteria for Adverse Events (CTCAE) standards, reliable information on PRO should not only improve symptom management but also allow for the reduction of emergency admissions and improve patients’ quality of life. However, early responsiveness to symptoms and presumably longer continuation of chemotherapy, as well as a potential benefit of follow-up integration of ePRO for symptom monitoring during routine cancer care, frequently involve patient-physician or patient–nurse specialist communication [9,10]. In addition, compliance rates and the use of symptom alerts seem to be enhanced by structured graphic displays on outcome reporting [3,4]. Several digital platforms are currently implementing the capture of ePRO to allow for the sharing of data with treatment teams or to apply automatic algorithms for alert notifications in a timely and structured manner if symptoms worsen [2,11,12]. The consilium care smartphone app continuously allows oncologists to monitor the progress of patients’ symptoms through visualized progression charts based on structured patient entries. In the case of severe symptoms that exceed a determined threshold, the app notifies the patient to contact the treatment center. Previous published breast cancer studies showed the potential of the app to stabilize daily functional activity and well-being of patients in collaboration with the physician [1]. In addition, more distinct symptom entries were received from those users who shared reporting with their physicians. The functionality and utility of 2 comparable app versions for collecting ePRO have also demonstrated that the request for a collaborative review of ePRO for shared reporting increases the number of data entries and potentially affects the ability to deal with the symptoms of illness [13]. Since clinical oncology strives for a standardized recording of adverse events, the congruence between doctor and patient should serve as an important indicator that patients’ self-reporting can enhance the quality of outcome data for the accuracy of clinician ratings and safety. This has the potential to reduce the problem of patients reporting high symptom severity while their clinicians note low toxicity grades. Further, it has the potential to identify challenges in effective patient-clinician communication regarding symptom experience, to stimulate the processes of recording and reviewing patient-reported symptoms, to facilitate consultation with oncologists, and to provide self-care algorithms for real-time interventions that reduce symptom severity [13].

In this study, we evaluated the efforts being made using the consilium care app in a cohort of patients with breast, colon, lung, or prostate cancer, as well as those with hematological malignancies, to demonstrate the reliability of electronically captured patient-reported symptom entries for shared reporting with the physician to detect critical symptoms in routine cancer care. For this study, we intended to demonstrate that a collaborative review of randomly selected patient-reported symptoms improves congruency of patient- and clinician-reported toxicity in patients receiving systemic anticancer therapy. In particular, we examined whether important and frequent symptoms, such as pain, fever, diarrhea, obstipation, nausea, vomiting, and stomatitis, can be described appropriately according to the CTCAE in order to potentially implement recommendations for mitigation.

## Methods

### Study Design

We conducted a multicenter, observational, noninterventional study. The protocol was approved by the competent regulatory ethics committee (KEK-ZH:2017-02028) and registered on ClinicalTrials.gov (NCT03578731). Patients with breast, colon, prostate, or lung cancer, as well as those with hematological malignancies, aged 18 years and older, and initiating adjuvant or neoadjuvant systemic therapy were eligible to participate after providing written informed consent. In addition, participants had to speak German and own a smartphone. Eligible participants were recruited consecutively and without preselection according to the recommendation of the local tumor boards in centers in Switzerland, Germany, and Austria.

### Objective

The primary objective of this study was to assess the level of agreement, κ, between symptom ratings by physicians at the time of the regular consultation and the ratings derived from the daily PRO between consultations. The level of agreement was analyzed in order to determine the reliability and utility of self-reported electronic symptom monitoring.

### Mobile App

To begin, patients downloaded the consilium care app (available for iOS and Android) and connected themselves via a quick response code to their study centers. For the patients’ convenience, a summary of diagnostic workup, treatment medication, and contact information of the respective treatment center was entered into the consilium care web app—the treatment team’s counterpart to the smartphone app.

**Figure 1:**
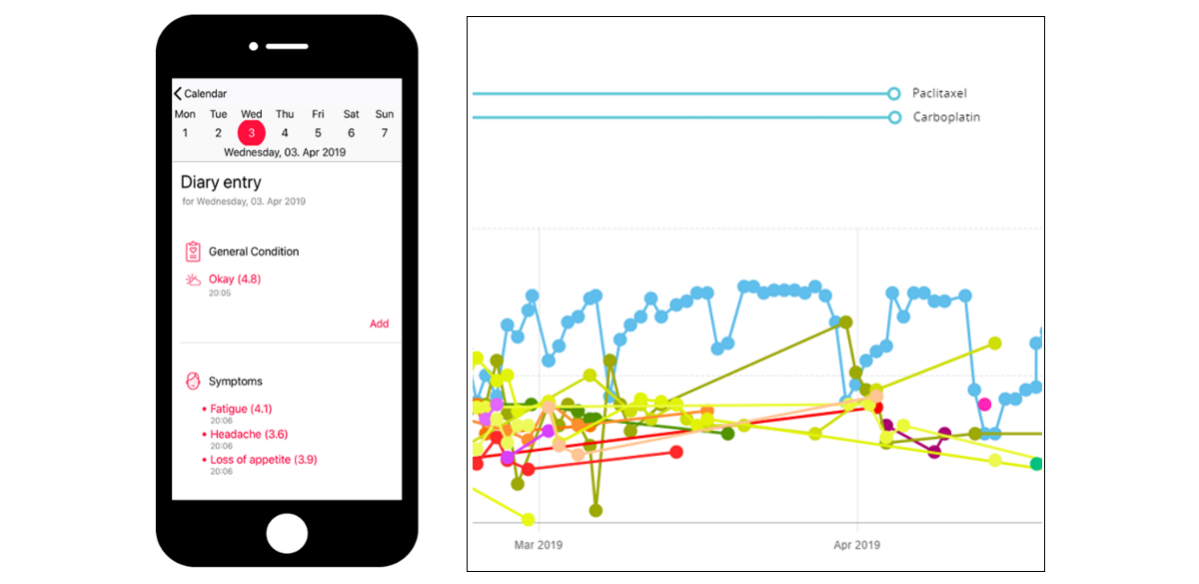
Entrance screen and a representative symptom history chart with indication of medication, well-being (blue graph), and various symptoms presented in different colors.

The app (Figure 1) facilitated the selection of well-being, symptoms, medication, and private notes. Symptoms, which were structured in groups according to organ systems, could be selected. The symptom entry display (52 distinct symptoms were available for which severity, onset, and duration could be indicated) was equipped with date and time stamps. Symptom severity, with descriptions based on the CTCAE, could be selected via a slider. The symptom history was displayed on a timeline with individual colors for each symptom. In addition, diary entries and information on diagnosis and therapy were indicated separately.

Patients were encouraged to capture data on well-being and symptoms on a daily basis. Recording usually started on the day of the therapy’s initiation or the change in therapy and continued through an observational period of 12 weeks. The app allowed the continuous recording of well-being and symptoms based on the CTCAE through use of virtual analogue scales. Definitions for CTCAE grades were displayed above the slider, with which the grade of the entry could be selected via the virtual analogue scales. The severity level of a symptom, as rated by physicians and patients, was measured on an ordinal scale, with 0 indicating the lowest possible degree of severity and 4 indicating the highest possible one. The history of recorded data was displayed and visualized in the form of a symptom progression chart. In the case of severe symptoms, patients were encouraged by push notifications to seek medical advice. In addition, patients recorded their well-being according to the Eastern Cooperative Oncology Group (ECOG) performance status via a slider, with possible impairments in daily functional activities being displayed. Information for self-care (derived from the Swiss Cancer League and the Sächsische Krebsgesellschaft) was provided to them via the app depending on the severity of symptoms upon data entry.

Functional data security was ensured by identification being made only possible through the patient’s ID. The data on the patient’s device were encapsulated in the app, and data exchange was encrypted with the patient ID. At the study center, personal data were kept strictly separate from the data collected by the app. Data matching was performed by using the patient ID.

### Collaborative Symptom Reviews

Patients were assigned to medical oncology visits every 3 weeks and invited for shared reporting and intended symptom review, which were preferably scheduled on days of therapeutic intervention. Some exceptions were made for reviews to be carried out over the phone. At the scheduled visit, the app was triggered to randomly select 2 patient-reported symptoms from the past 20 days. A first measurement of congruence (symptom 1) was performed on a symptom that was entered 2 to 3 weeks (14 to 21 days) before the actual consultation, whereas a second measurement (symptom 2) was performed on a symptom that was entered within the previous week (1 to 7 days). Patients and physicians were then prompted to perform a detailed, shared review of these symptoms in order to focus on the collection and appropriate interpretation for symptom severity grading. Up to 4 such reviews were planned per patient, including 2 electronic symptom entries per review.

### Questionnaire

At the end of the observational period, participants were asked to complete a questionnaire on paper regarding the usability and usefulness of the app to clarify quality of care and the relationship between the patient and physician during the course of treatment. To this end, a 5-point Likert scale was used, with a rating from 1 (disagree) to 5 (agree very strongly).

### Sample Size

We calculated the sample size on a 5% significance level to test the level of agreement, κ=0.5, between 2 raters (ie, fair to good agreement) with a precision of 0.1 on each side of 156 patients. In order to estimate κ with the necessary precision within these subgroups, we included at least 170 patients with breast cancer and 170 patients with colon cancer. We anticipated a difficultly in recruiting the same number of patients with lung cancer or prostate cancer due to their lower prevalence. Thus, the aim was to include 130 patients with either lung cancer (not fewer than 50) or prostate cancer and 130 patients with hematological malignancies. We planned to enroll a total of 600 patients, as we expected 15% to 20% of enrolled patients to discontinue participation (dropout) early.

The originally planned study population size for the entire study cohort was 600. The study duration was estimated to be about 3 years, starting in March 2018. In autumn 2020, only about one-third of the planned study patients were recruited, and the sponsor decided to prematurely terminate the study on October 11, 2020, due to insufficient recruitment. Despite the continuous opening of many study sites beginning 2018, due to the present recruitment rate and the ongoing COVID-19 situation, the planned number of 600 patients was unachievable.

### Statistical Analysis

Descriptive statistics included mean and SD for continuous variables, and numbers and percentages of total for categorical variables. For statistical analysis, the associations between physicians’ and patients’ ratings were visualized by plots. Multiple ratings for patients were included and accounted for by the analysis. For the quantification of levels of agreement, Cohen kappa (κ) values were calculated with squared weights. κ values are reported with 95% CIs. These CIs were based on 1000 bootstrap samples. According to Landis and Koch [14], values for κ were characterized as follows: <0, no agreement; 0 to 0.20, slight agreement; 0.21 to 0.40, fair agreement; 0.41 to 0.60, moderate agreement; 0.61 to 0.80, substantial agreement; and 0.81 to 1, almost perfect agreement. All analyses were carried out with R version 4.0.2 (The R Foundation for Statistical Computing) [15], and Excel R Markdown was used for dynamic reporting.

## Results

### Baseline Characteristics

Between February 2018 and October 2020, 223 patients (190 female and 33 male) with cancer (170 breast, 19 lung, 15 colon, 7 prostate, and 12 hematological [B cell] malignancies) were included using the consilium care app. Among them, 181 patients (158 women and 23 men; age at therapy start: mean 54.4 years, SD 12.1) had performed at least 1 validated review with the treating physician. About half of the 181 patients who used the consilium care app were treated in an adjuvant setting (vs neoadjuvant). Fewer than one-third (51/181, 28.2%) of the patients received treatment for advanced disease with noncurative intention. In total, 27 distinct chemotherapeutic agents in 17 different chemotherapy regimens were administered, including antihormones, CDK4/6 inhibitors, and immunotherapies.

Due to the lack of appropriate accrual within the context of the COVID-19 pandemic, premature closing of the study, and other issues, 42 patients included could not perform a minimum of 1 intended review. In addition to this, 7 patients were not evaluable due to the premature study termination, 10 patients did not enter a sufficient number of symptoms, and another 14 patients were not evaluable due to technical issues. Only 3 patients withdrew their informed consent. Baseline characteristics are displayed in Table 1, and an overview flow chart of the patient enrollment is available in Multimedia Appendix 1.

**Table 1:** Baseline characteristics.

Characteristic		Value (N=181)
**Primary tumor, n (%)**		
	Hematological	9 (5.0)
	Breast	142 (78.5)
	Colon	11 (6.1)
	Lung	13 (7.2)
	Prostate	6 (3.3)
**Sex, n (%)**		
	Female	157 (86.7)
	Male	23 (12.7)
	N/A^a^	1 (0.6)
	Age at start, mean (SD)	54.4 (12.1)

^a^N/A: not applicable.

### Agreement Levels

A total of 181 patients underwent at least 1 intended symptom review for this analysis. From a subset of 110 patients (60.8%), more than 2 collaborative symptom reviews of patients with their physicians were available for analysis. For the analysis of the first symptom agreement levels (across all multiple ratings per patient), there were 497 (first rating) reviews available for analysis, while for the second symptom agreement levels, 483 reviews (second rating) were available.

**Figure 2:**
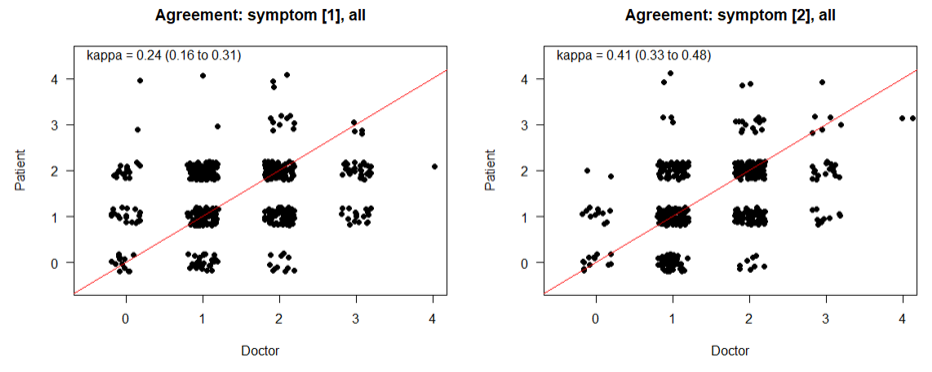
Estimations of agreement levels between physicians’ and patients’ observations for the first and second symptom. diarr: diarrhea; fev: fever; obstip: obstipation; stomat: stomatatis; vomit: vomiting.

An estimation of general agreement levels between physicians’ and patients’ observations in the first symptom (defined as recorded 14 to 21 days before the review) revealed a fair congruency of κ=0.24 (95% CI 0.16-0.33), while for the second most recent symptom (defined as being recorded 1 to 7 days before), the value rose to κ=0.41 (95% CI 0.34-0.48; Figure 2).

**Figure 3:**
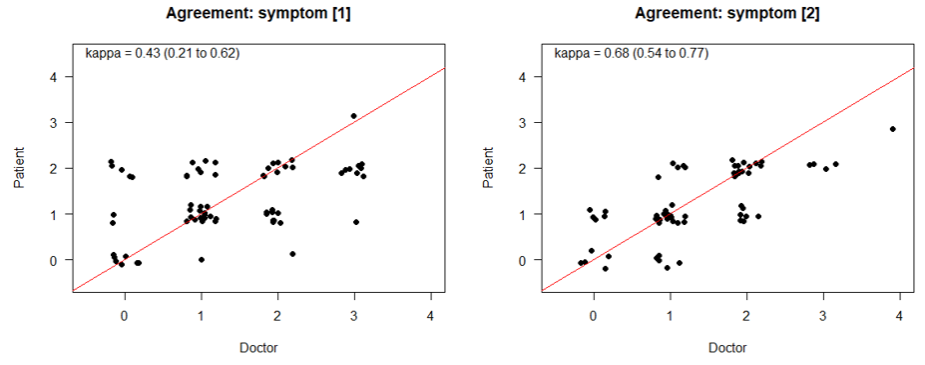
Estimations of agreement levels between physicians’ and patients’ observations for specific symptoms.

Analysis of the levels of agreement in subgroups of the specific symptoms, including pain, fever, diarrhea, obstipation, nausea, vomiting, and stomatitis, revealed a higher congruency between the patient and physician estimate (symptom 1: κ=0.43, 95% CI 0.21-0.62; symptom 2: κ =0.68, 95% CI 0.54-0.77; Figure 3). Whether this observation was due to a different perception of clinical relevance and frequency of these symptoms or to a clearer description, as 5 of the 7 symptoms were associated with objectifiable values in their definition (eg, fewer than 4 loose stools per day) at some point, remains unclear.

**Figure 4:**
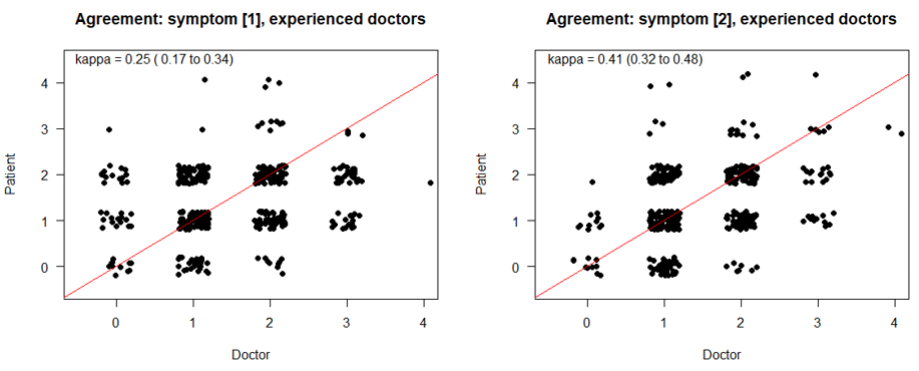
Estimations of agreement levels between physicians’ and patients’ observations for experienced physicians.

Next, we evaluated the levels of agreement in the subgroup of physicians with at least 10 ratings. The distribution of rating frequencies revealed large differences; of the 29 participating in this study, 9 physicians performed 10 or more ratings. These were considered experienced raters and were included in the subsequent assessments. For the analysis, there were 417 observations for symptom 1 (first rating) and 405 observations for symptom 2 (second rating). Again, multiple ratings per patient were included. As shown in Figure 4, a fair congruency between patient and physician estimates was present for those considered experienced (≥10 ratings; symptom 1: κ =0.25, 95% CI 0.17-0.34; symptom 2: κ=0.41, 95% CI 0.33-0.49). Compared to all physicians’ (experienced and less experienced) ratings for symptom 1 (κ=0.24) and symptom 2 (κ=0.41), the agreement levels hardly differed, indicating that congruency was more likely affected by timing and symptom description than the physicians’ particular skills.

**Figure 5:**
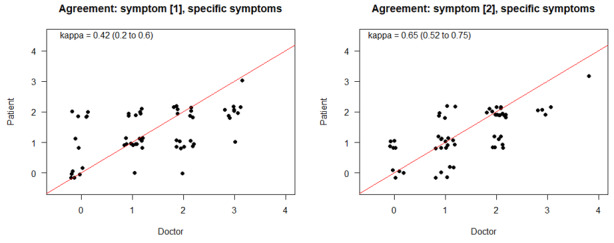
Estimations of agreement levels of experienced physicians specifically for the most frequent symptoms.

Similar results of congruency as those seen in the specific symptoms displayed in Figure 2 were obtained for the most frequent symptom as rated by experienced physicians (>10 ratings; symptom 1: κ=0.42, 95% CI 0.18-0.62; symptom 2: κ =0.65, 95% CI 0.5-0.75; Figure 5). The most frequently captured symptoms were fatigue, hot flashes, sleep disorder, headache, and taste disorder.

The levels of agreement with respect to time intervals between the date of collaborative review and the date of symptom entry within the previous week did not reveal a significant difference (days 1-3: κ=0.40; days 4-7: κ=0.39; overall days 1-7: κ=0.41). For the rating of symptoms entered 15 to 21 days prior to the review, a significant lack of congruency was noted (κ=–0.15). This finding indicated that patients recalled symptoms and their severity much better if they occurred more recently. For future studies, a collaborative review of a symptom from the recent past may be considered sufficient to demonstrate the accuracy of the electronic symptom recording in general, particularly for distinct and frequently occurring symptoms. Although this observation might require confirmation in a subsequent study, the idea of recent-past symptom validation (less than 7 days) might be applicable in real-world cancer care, clinical trials, or pay-for-performance models [16]. Furthermore, we noted a moderate increase of congruence between ratings from week 3 (first rating) to week 9 (third rating) in our approach (symptom 1: κ=0.23 vs κ=0.29; symptom 2: κ=0.36 vs κ=0.41), indicating a potential training effect in patients and physicians. The quality of ratings neither appeared differently with regard to light or moderate symptoms (CTCAE grade ≤ 2) nor in comparison to severe symptoms, defined as CTCAE grade >2 (κ=0.13 vs κ=0.11), which is important in cases of early-intervention clinical practice. Congruency of symptom reporting according to the review of the second symptom was similar for breast (396 reviews; κ=0.39), lung (30 reviews; κ=0.45), and colon cancer (23 reviews; κ=0.51), as well as hematological malignancies (20 reviews; κ=0.49). For prostate cancer, there was an almost perfect congruency (12 reviews; κ=0.82) although the low number of reviews had to be considered with regard to statistical significance. The subgroup analysis for age and gender showed overall congruency levels of κ=0.50 for older (>65 years; 99 reviews; κ=0.50;) and younger patients (<65 years; 380 reviews; κ=0.38), as well as for female (435 reviews; κ=0.40) and male (44 reviews; κ=0.49 for) patients.

### Well-being and Symptoms

**Figure 6:**
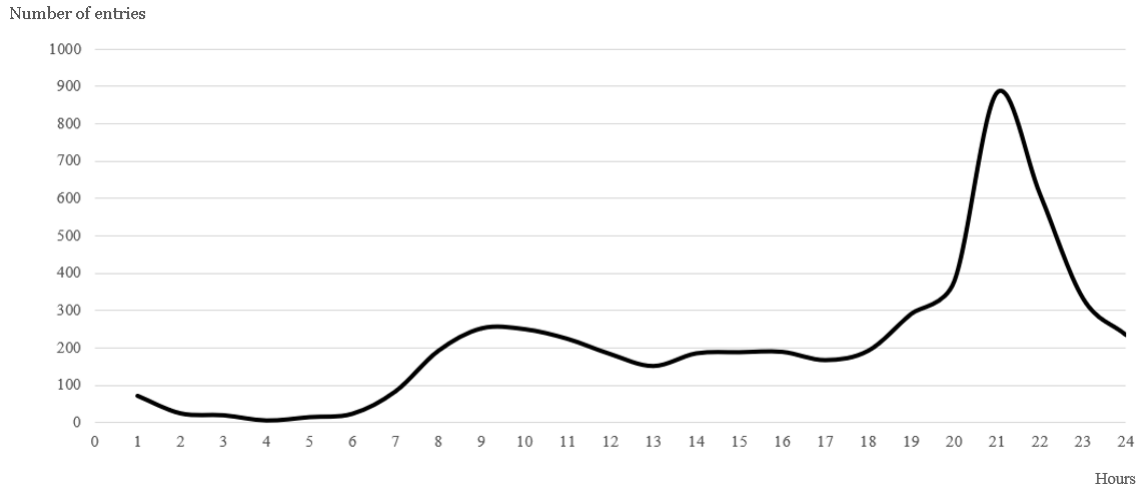
Circadian distribution of well-being entries (y-axis) over 24 hours (x-axis).

Regarding well-being, 4762 data entries were derived from 210 evaluable patients during the observation period. Patients reported their well-being almost every single day and in a classical circadian rhythm (Figure 6). Because well-being was reported independently of the underlying diagnosis or symptoms, we assumed that this indicated a pattern of app use. Users preferred to use the app in the morning and also used it during the evening hours. Therefore, a circadian pattern of symptom reporting seemed to be favored. The degree to which the app’s functions (eg, occasional push notifications, design features, tips for self-care, or effects of collaborative review and shared reporting) affected data entries remains unclear, as this evaluation was not addressed.

Overall, 210 patients generated a large absolute number of 42,142 electronically reported symptoms and side effects, suggesting easy handling of the app for an effective symptom history insight. Given the observational period of 84 days, this resulted in an average number of 2 to 3 entries per patient and day. The most commonly reported symptom was fatigue, which was indicated significantly more often in the breast cancer and lymphoma groups (data not shown) compared to other cancer entities. Due to the heterogeneity of drugs and limited information on dosage, a potential association of symptoms with the respective cancer type, medication, or regimen, could not be performed sufficiently. However, more than 32.59% (13,734/42,142) of all data entries affected usual activities of daily living and symptoms such as pain/discomfort (8370/42,142, 19.86%), self-care (3475/42,142, 8.24%), anxiety/depression (1458/42,142, 3.45%), and mobility (431/42,142, 1.02%), all of which potentially represent components of the 5-level EQ-5D questionnaire.

### Unplanned Consultations and Serious Adverse Events

Although fewer than 18.2% (33/181) of the participants with solid cancer (breast, colon, lung, prostate) required unplanned consultations or emergency services due to treatment-related side effects and toxicities, more than twice this proportion (4/9, 44%) was recorded in patients with lymphoma, mostly attributed due to fatigue and fever. An association with a possible benefit from app use cannot be made, as data from a matched analysis (age, cancer type, therapy) of patients from 2 larger participating cancer centers indicated only a nonsignificant decline in these events (data not shown). Importantly, no serious adverse events related to the use of the app were recorded during the entire study period.

### Usability and Usefulness of the App

Questionnaires from 171 patients included were available for the rating of the app at the time of this survey. Six patients died due to cancer progression during the study, from whom surveys were not available for analysis. A utility analysis could not be conducted on 16 patients, as they were not correctly included into the study, withdrew informed consent, had technical problems, or lacked a sufficient number of data entries. The results are displayed in Multimedia Appendix 2.

## Discussion

The systematic electronic recording of PRO by smartphone has not yet been extensively explored in cancer treatment. Previous studies indicate that the range of measures used and symptoms captured seem to vary greatly across studies, and that, regardless of the concordance metric employed, the reported agreement between clinician-based CTCAE and PRO seems to be moderate, at best [17]. In one study that retrospectively applied CTCAE patient language adaptations, including the Symptom Tracking and Reporting system, to assess specific symptoms, extracted clinician- and patient-reported adverse event ratings were considered poor to moderate, at best, when the applied rating sources for each of the adverse events were compared [18]. In an attempt to improve these differences, we explored integrating ePRO and clinician reporting with a standardized, shared review process, according to CTCAE criteria with adapted patient-oriented language by testing the level of agreement between the patients’ and physicians’ judgment on the severity of patient symptoms with 3 weekly reviews of randomly selected symptoms at any severity grade.

Overall, we found fair agreement for long-lasting symptoms, whereas for the more recent symptoms (defined as those recorded 1-7 days earlier), the degree of agreement in symptom reporting between the patient and physician was moderate and comparable to results from a study in early breast cancer [19]. However, the congruence between patients and physicians gained substantial reliability when analyses on levels of agreement in subgroups of the specific symptoms (ie, pain, fever, diarrhea, obstipation, nausea, vomiting, and stomatitis) were performed and also in an identical manner to that in the most frequently occurring symptoms, including fatigue, hot flashes, sleep disorder, headache, and taste disorder. Together, data entries from these symptoms covered about 50% of all recorded symptom-related entries during this study. As patients obviously recalled recent symptoms more clearly, the high trustworthiness of symptom rating could be sufficiently proven by 1 review in this context. Congruency of rating seemed to be independent of the reviewers’ experience, and no outlier result in congruency of symptom reporting could be demonstrated for any specific patient cohort, indicating the potentially broad acceptance and use of such an approach. Additionally, no differences in symptom congruency were noted with respect to light or more severe symptom grading.

Compliance for the use of the consilium care app was high as evidenced by the high number of 2 to 3 data entries per patient and by the response from questionnaires, and was found to be comparable with results from other studies that used more standardized questionnaires for different devices [20]. In a recent study, patients were invited to complete the European Organization for Research and Treatment of Cancer (EORTC) Core Quality of Life questionnaire (QLQ-C30) and cancer site–specific modules before each visit on tablets or computers in the hospital or at home. An adequate compliance (at least 66% of health-related quality of life assessments were completed) was demonstrated for the cohort of breast cancer (96%), colorectal cancer (98%), and lung cancer (91%) [21], which we consider comparable to the results of our study.

In one study that administered weekly PRO from the National Cancer Institute’s PRO-CTCAE item library (for symptoms such as pain, nausea, and diarrhea) via mail or telephone and assessed them by using a 5-point ordinal verbal descriptor scale and via PRO questions about physical performance (ECOG) and financial toxicities (The Comprehensive Score for Financial Toxicity [COST-FACIT] questionnaire), it was found that most patients agreed that weekly reporting was a favored frequency for ePRO questionnaire administration in the context of advanced and metastatic cancer treatment [22]. However, during a more complex or intensive treatment phase, a more frequent (even daily) assessment of more than 8 symptoms might be well regarded and positively associated with an increased use of educational materials about home symptom management. In another trial, almost 40% of patients (particularly older patients and those living in rural areas) chose to use an automated telephone interface rather than a web interface or preferred personal contact in the case of severe symptoms affecting cognitive or sexual dysfunction [23]. Web-based, guided, self-help interventions can provide clinically meaningful improvements in quality of life; however, producing a meaningful effect might require punctual psychological interventions [24]. Although no such findings were apparent in our study, following advice and using tips for timely self-care and compliance remains challenging for patients and caregivers. The consilium app contains 20 tips for the most common symptoms. In personal communication with patients, it was suggested that this opportunity of self-help intervention should be linked to the appropriate symptom or grade, as patients perceived this to be a component of personalized medicine [25,26].

There were potential limitations to this study. The frequency of the completed symptom reviews varied between the 3 German-speaking countries conducting the trial, most patients were suffering from breast cancer, and the study was not randomized, which precluded analysis in regard to the effects of empowered self-care and the potential impact on unplanned consultations. Statistical limitations evolved from the data set when there were multiple observations per patient; thus, observations could not be considered independent. Furthermore, there were limitations to the interpretation of Cohen κ values. In this study, we used magnitude guidelines proposed by Landis and Koch [14] to describe levels of interrater reliability; however, other guidelines exist, such as those of Fleiss [27]. Because of the ongoing debate about the correct description of κ values, the interpretation we employed can still be subject to scrutiny. Importantly, due to the lack of appropriate accrual in the context of the COVID-19 pandemic, the trial was ended prematurely.

In summary, we demonstrated that a shared monitoring and review process to assess symptoms between patients and physicians has the potential to improve the quality of future patient self-reporting. Our study indicated that the integration of ePRO into oncological clinical research and continuous clinical practice should leverage monitoring of side effects and symptom management [28,29] using the rapidly developing digital mobile and sensor technologies, which can provide more objective measures and facilitate the active and passive collection of detailed, personalized data.

## Appendix

Multimedia Appendix 1 Overview flow chart of the patient enrollment.

Multimedia Appendix 2 Results of the questionnaire about the usability and usefulness of the app.

## Abbreviations

COST-FACIT: The Comprehensive Score for Financial Toxicity
CTCAE: Common Terminology Criteria of Adverse Events
ECOG: Eastern Cooperative Oncology Group
ePRO: electronic patient-reported outcomes
EORTC: European Organization for Research and Treatment of Cancer
PRO: patient-reported outcomes
