# Supplementary figures and images for: The Effect of Collaborative Reviews of Electronic Patient-Reported Outcomes on the Congruence of Patient- and Clinician-Reported Toxicity in Cancer Patients Receiving Systemic Therapy: Prospective, Multicenter, Observational Clinical Trial
Source: J Med Internet Res. 2021 Aug 5;23(8):e29271. doi: 10.2196/29271 (PMC8380582; doi:10.2196/29271)

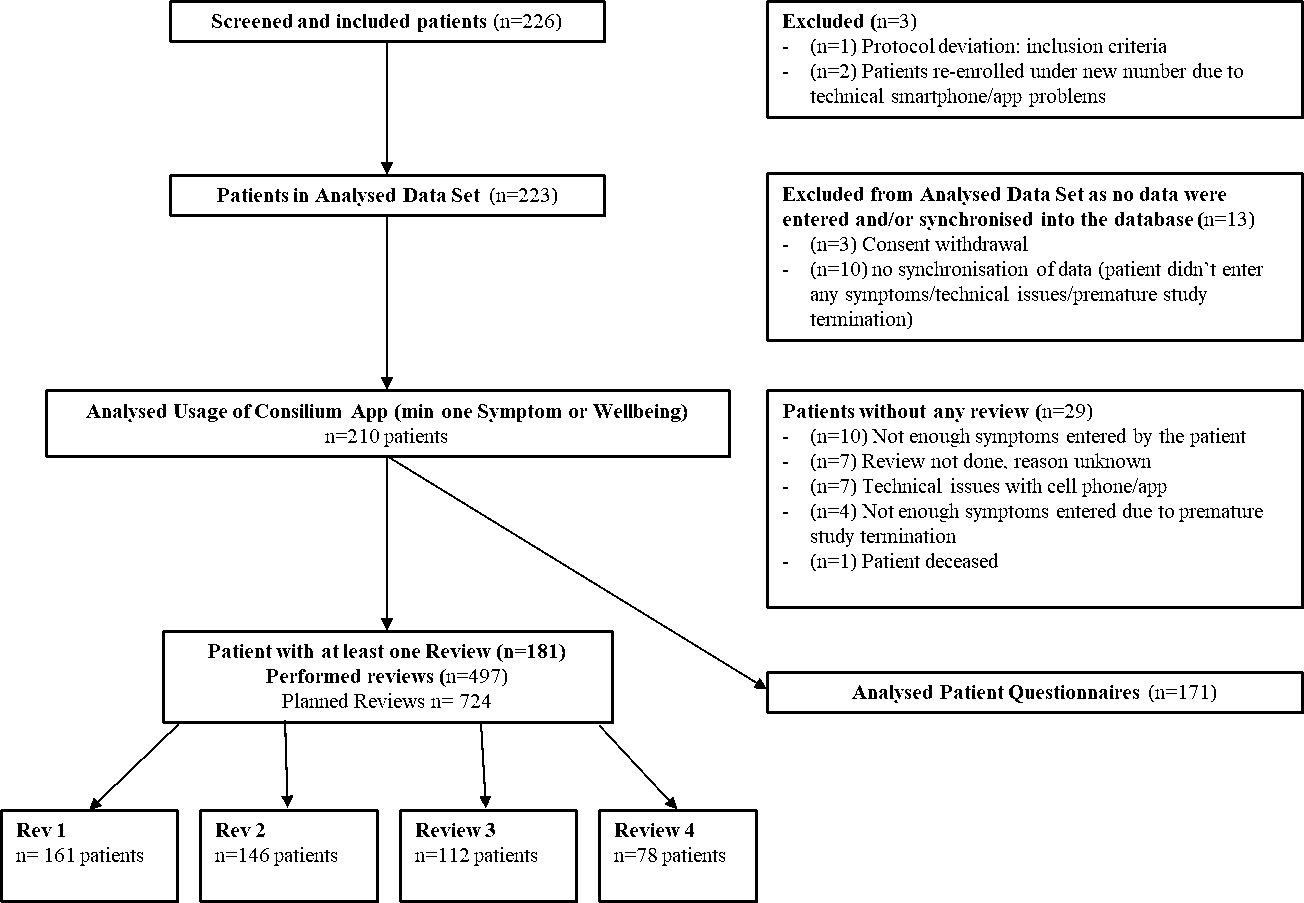

Supplement: Multimedia Appendix 1 [file jmir_v23i8e29271_app1.png]

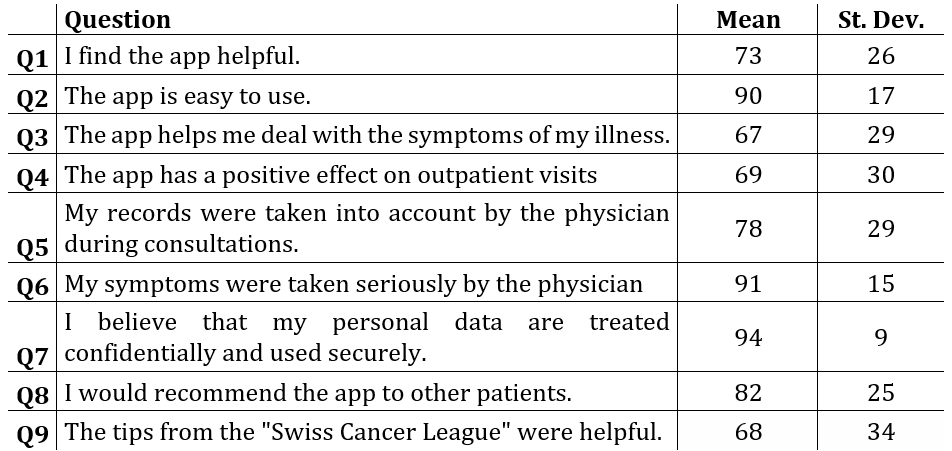

Supplement: Multimedia Appendix 2 [file jmir_v23i8e29271_app2.png]
